# Evaluating the forced oscillation technique in the detection of early smoking-induced respiratory changes

**DOI:** 10.1186/1475-925X-8-22

**Published:** 2009-09-25

**Authors:** Alvaro CD Faria, Agnaldo J Lopes, José M Jansen, Pedro L Melo

**Affiliations:** 1 Department of Physiology, Biomedical Instrumentation Laboratory, State University of Rio de Janeiro, Rio de Janeiro, Brazil; 2 Faculty of Medical Sciences, Pulmonary Function Laboratory, State University of Rio de Janeiro, Rio de Janeiro, Brazil; 3 Department of Physiology, BioVasc Research Laboratory, State University of Rio de Janeiro, Rio de Janeiro, Brazil

## Abstract

**Background:**

Early detection of the effects of smoking is of the utmost importance in the prevention of chronic obstructive pulmonary disease (COPD). The forced oscillation technique (FOT) is easy to perform since it requires only tidal breathing and offers a detailed approach to investigate the mechanical properties of the respiratory system. The FOT was recently suggested as an attractive alternative for diagnosing initial obstruction in COPD, which may be helpful in detecting COPD in its initial phases. Thus, the purpose of this study was twofold: (1) to evaluate the ability of FOT to detect early smoking-induced respiratory alterations; and (2) to compare the sensitivity of FOT with spirometry in a sample of low tobacco-dose subjects.

**Methods:**

Results from a group of 28 smokers with a tobacco consumption of 11.2 ± 7.3 pack-years were compared with a control group formed by 28 healthy subjects using receiver operating characteristic (ROC) curves and a questionnaire as a gold standard. The early adverse effects of smoking were adequately detected by the absolute value of the respiratory impedance (*Z4Hz*), the intercept resistance (*R0*), and the respiratory system dynamic compliance (*Crs, dyn*). *Z4Hz *was the most accurate parameter (Se = 75%, Sp = 75%), followed by *R0 *and *Crs, dyn*. The performances of the FOT parameters in the detection of the early effects of smoking were higher than that of spirometry (p < 0.05).

**Conclusion:**

This study shows that FOT can be used to detect early smoking-induced respiratory changes while these pathologic changes are still potentially reversible. These findings support the use of FOT as a versatile clinical diagnostic tool in aiding COPD prevention and treatment.

## Introduction

Chronic Obstructive Pulmonary Disease (COPD) is widely recognized as a leading and growing cause of mortality worldwide and as a major global public health problem [1]. Unfortunately, the diagnosis of COPD is usually obtained only in late stages, when respiratory function is already impaired. Due to the high prevalence and high medical costs associated with COPD, early identification and treatment of these patients is important in order to avoid severe and expensive stages of this disease [2]. Another reason for early identification of COPD is the progressive and irreversible nature of the disease. Recently, it was observed that the deterioration in pulmonary function associated with the development of COPD is directly related to the duration of the smoking habit and to the number of pack-years consumed [3,4].

The respiratory effects of smoking are usually evaluated by using respiratory flows and volumes obtained by spirometry. However, not all modifications in respiratory mechanics are always detected by spirometry [5]. Moreover, some patients are not able to reliably perform spirometry since it requires good subject co-operation and maximal effort [6]. Although experts agree it would be desirable to have some test or intervention that would allow early identification of COPD, enabling earlier treatment or prevention of more severe stages of the disease, there is tremendous controversy as to whether spirometry is that test. In fact, there is agreement in the literature that new measurement technologies that are able to detect COPD in early stages would contribute to decreasing medical and economic burdens [2].

The Forced Oscillation Technique (FOT) offers a simple and detailed approach to investigate the mechanical properties of the respiratory system. This method characterizes the respiratory impedance and its two components, respiratory system resistance (*Rrs*) and reactance (*Xrs*). These parameters are usually measured at various frequencies by means of small pressure oscillations (about 2 cmH_2_O) superimposed at the mouth during spontaneous breathing. The method is simple and requires only passive co-operation and no forced expiratory maneuvers. Another important advantage, particularly in pathophysiological research, is that FOT can be used to provide information on the mechanical characteristics of the respiratory system that are complementary to spirometry [7-11]. Impulse Oscillation Systems (IOS) [12] and the Airflow Perturbation Device (APD) [13,14] are similar techniques that also provide complementary information. FOT has been validated over a period of 30 years [8], whereas IOS and APD are relatively new developments.

The FOT has the potential to greatly increase our knowledge regarding the pathophysiology of smoking, as well as to help in the clinical diagnosis of early smoking-induced respiratory alterations. This technique has been applied previously to obtain a detailed analysis of respiratory mechanics in smokers compared with non-smokers [5,15,16], as well as for comparisons among non-smokers, former smokers and current smokers [17]. However, there are few data evaluating the clinical performance of the FOT indices in the detection of early smoking-induced alterations in respiratory system resistance and reactance. It is known that respiratory system resistance increases are associated with airway obstruction and ventilation non-homogeneity. On the other hand, more negative reactance values are related to reduced dynamic compliance and increased work of breathing. These alterations are closely associated with the early deleterious effects of smoking, and the posterior diagnostic of clinically relevant COPD [18].

It is not known if subjects presenting early changes associated with low tobacco consumption will actually progress to clinically significant COPD later on. In fact, a follow-up study of those smokers would be necessary to answer this question. However, this kind of study is not easy to conduct due to ethical issues. A recent consensus recommended that all smokers, including those who may be at risk for COPD as well as those who already have the disease, should be offered the most intensive smoking cessation intervention feasible [19]. This consensus also recommended that patients should be identified as early in the course of the disease as possible, contributing to prevent smoking uptake and maximize cessation [19].

Therefore, the purpose of this paper is to evaluate the ability of FOT to detect the respiratory effects of smoking. We ask two basic questions: (I) Whether FOT parameters can detect the harmful respiratory effects of smoking while they are still potentially reversible, before the patients reaches the clinical diagnosis of COPD, and (II) if so, whether FOT is more sensitive than spirometry at detecting the earliest signs of respiratory system malfunction.

## Methods

### Subjects

This study was designed as a case-control study, and was approved by the Ethics Committee of the State University of Rio de Janeiro. Informed consent was obtained from all volunteers before inclusion in the study. Healthy control subjects with normal spirometry who had never smoked, as well as smoking subjects who were on no regular medications and had no allergic, respiratory, cardiovascular, gastrointestinal, renal or neurological symptoms were recruited among people working in the laboratory and from the State University of Rio de Janeiro area. All subjects had stable health for at least four consecutive weeks and signed written informed consent. Baseline data, including age, height and weight, were obtained from each subject at the time of the procedures.

The amount of tobacco smoked and the duration of smoking were quantified using the number of pack-years [20]. Verbanck et al. [21], using the multiple breath washout test, showed that the earliest signs of small airway malfunction in smoking patients are seen from 10 pack-years onwards. Therefore, we studied smokers with tobacco consumptions near 10 pack-years who were recruited from both university personnel who smoke and from patients who visited the smoking cessation clinic of our university hospital. All smokers were current smokers. These volunteers had been instructed to abstain from smoking for at least two hours before the testing in order to avoid acute effects of tobacco use on lung function [22].

### Equipment

Total respiratory resistance and reactance were measured using a forced oscillation system, which has previously been described in detail [23,24]. The system was a standard multifrequency FOT test system operating in the most commonly used frequency range [7,10]. Measurements were conducted in conformity with the recommendations issued by a task force of the European Respiratory Society [10]. Briefly, small amplitude pressure variations from 4-32 Hz generated by a loudspeaker were applied at the mouth using a mouthpiece. The pressure input was measured with a Honeywell 176 PC pressure transducer (Honeywell Microswitch, Boston, MA, USA), and the airflow was measured with a screen pneumotachograph connected to a similar pressure transducer with a matched frequency response. These signals were digitized at a rate of 1024 Hz for periods of 16 s by a personal computer. Usually a sampling frequency of 128 Hz is used when the maximum frequency is 32 Hz. In the present study, a higher sampling frequency was used in order to provide a detailed visual description of the artefactual events, such as swallowing or glottis closures, on the flow and pressure signals. As recommended [10], these signals are displayed on the screen during the measurement. The fast Fourier transforms (FFT) of the digitized signals were computed using blocks of 4096 points with 50% overlap. Overlapping was used in order to increase the number of independent data blocks and reduce random errors. Three measurements of 16 s each were made, and the result of the test was calculated as the mean of these measurements.

Resistive impedance data were subjected to linear regression analysis over the frequency range from 4 to 16 Hz. Previous studies from our group [18,25] showed that most of the changes due to airway obstruction are observed over this frequency range. The resistive impedance at 0 Hz (*R0*) was extrapolated from this analysis. This parameter is related to the total resistance of the respiratory system and is usually used as an index of airway obstruction [26]. The mean resistance (*Rm*), sensitive primarily to airway caliber [8], was also calculated for the frequency range from 4 to 16 Hz. The slope of the resistive component of the respiratory impedance (*S*) (which is associated with respiratory system homogeneity [17,27]) was also obtained from the analysis. Negative values of this parameter reflect abnormal patterns of ventilation distribution. Previous works reported *S *values near zero in healthy subjects [23,24,28].

The properties of energy accumulation were characterized by the mean reactance (*Xm*), a property usually related to respiratory system non-homogeneity [29]. This parameter was calculated based on the entire studied frequency range (4 to 32 Hz). These properties were also characterized by both the resonance frequency (*fr*), which is defined as the frequency at which the *Xrs *equals zero, and the respiratory system dynamic compliance (*Crs, dyn*), which was estimated using the *Xrs *at 4 Hz (*Crs, dyn *= -1/(2πf*Xrs*)) [30]. The same frequency was used to evaluate the absolute value of respiratory impedance (*Z4Hz*). This variable represents the total mechanical load of the respiratory system [7-10], and is associated with the necessary work to promote the movement of air in the respiratory system. In the present work, Z values measured at 4 Hz mainly describes the resulting effect of the total resistance and respiratory system compliance.

### Protocol

**Figure 1:**
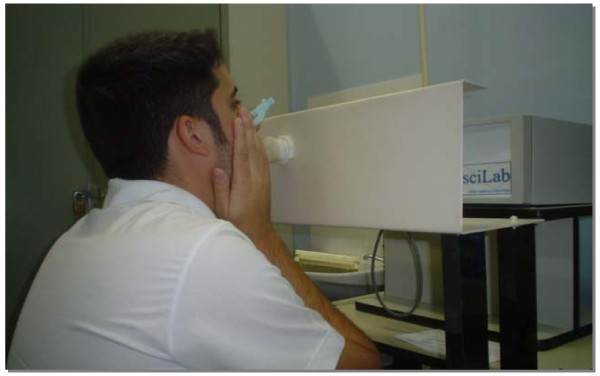
**Picture describing the forced oscillation measurements in a subject**.

To perform the FOT analysis, the volunteer remained in a sitting position, keeping his or her head in a normal position and breathing at functional residual capacity through a mouthpiece. During the measurements, the subjects wore a nose clip and firmly supported their cheeks and sub-mandible tissue with their hands [10,18,25] (Figure 1).

The validity of the data was measured by computing the coherence function, which evaluates the signal-to-noise ratio. This function is the equivalent in the frequency domain of the correlation coefficient used in the time domain. In order to minimize the effect of noise associated with the breathing signal, only values with a coherence function of 0.9 or more were considered adequate [16,26,31]. Any time the computed coherence was less than this threshold, the maneuver was considered invalid and the exam was repeated. Whenever adequate coherence measurements could not be obtained according to these criteria, the patient was excluded from the study [18,25]. The data acquisition started 30 seconds after the beginning of the exam in order to allow the volunteer to be accommodated to the equipment, and to guarantee the regularity of the respiratory pattern during the exam [10].

Measurements of Forced Vital Capacity (FVC), Forced Expiratory Volume for the first second (FEV_1_), FEV_1_/FVC, and the ratio of Forced Expiratory Flow (FEF) between 25% and 75% of FVC to FVC (FEF/FVC) were obtained for patients in a sitting position using a closed circuit spirometer (*Vitrace VT-139; Pro-médico, Rio de Janeiro, Brazil*). These parameters were presented as raw data and percentiles of the predicted values (%). Predicted values for spirometry were obtained from Knudson et al. [32] and Pereira et al. [33]. Forced expiratory maneuvers were repeated until three sequential measurements were obtained. The indices studied were those obtained through the better curve, which was selected based on the higher value of FEV_1 _plus FVC. Quality control of spirometry is given by the ATS criteria, with the software allowing the detection of non-acceptable maneuvers.

### Statistics

The volunteers were stratified, and when the achieved data presented a statistically normal distribution, smokers and controls were compared using a Student's *t*-test. A non-parametric test (Mann-Whitney U Test) was applied when the data did not present a normal distribution. These analyses were performed using STATISTICA 5.0 software. The results are presented as mean +/- standard deviation. A p value of less than 0.05 was considered statistically significant.

The number of pack-years was calculated by multiplying the mean number of packs (one pack = 20 cigarettes) consumed daily by the number of years that the subject had their smoking habit [20]. These data were collected via a questionnaire, which was interviewer-administered. Although questions on tobacco consumption may not precisely quantify respiratory changes, it is widely known that smoking introduces inflammation and narrowing of peripheral airways. Therefore, the presence the smoking habit was used as a reference in this case-control study to separate the two studied groups. The performance of the FOT indices in the detection of smoking-induced respiratory alterations in the studied sample was evaluated by means of receiver operating characteristic (ROC) curves [34,35], This method is able to identify the optimal cut-off point that discriminated most efficiently between the absence and presence of early respiratory changes in the studied sample. Analysis of ROC curves is performed by plotting sensitivity versus 1-specificity for each possible cut-off level. This way, the larger the area under the curve (AUC), the more valid the diagnostic test is. According to the literature, ROC curves with AUCs between 0.50 and 0.70 indicate low diagnostic accuracy, AUCs between 0.70 and 0.90 indicate moderate accuracy, and AUCs between 0.90 and 1.00 indicate high accuracy [34,36]. Goedhart et al. [37] considered 0.7 to be a good cut-off value for a useful discriminator for clinical use. In the present study, we considered 0.75 to be the minimum value of the AUC for adequate diagnostic accuracy. The ROC curves were constructed using MedCalc 8.2 (Medicalc Software, Mariakerke, Belgium).

The comparison of the performance of FOT and spirometry to identify early respiratory alterations in the studied sample was conducted considering the AUC obtained from these two techniques, following the theory described by Metz [38]. MedCalc 8.2 was used in these comparisons. The values of sensitivity, specificity, and AUC were obtained based on the optimal cut-off point, which was chosen in order to balance the values of sensitivity and specificity [34].

The required sample size was calculated in order to allow for the comparison of a clinically useful area (AUC ≥ 0.75) with a null hypothesis value (AUC = 0.5, meaning no discriminating power). This was calculated using the software MedCalc version 8.2, and assuming Type I and Type II errors of 20% [39]. The minimal required sample size resulted in 24 volunteers in each group.

## Results

Although the minimal sample size was 24 subjects, in order to reduce statistical errors even more, we evaluated 28 volunteers in each group. The age distribution, physical characteristics and spirometric parameters of the studied subjects are summarized in Table 1. The biometric characteristics of the two studied groups were well matched, and there were no significant differences between the groups. Smoking patients presented non-significant reductions in spirometric parameters.

**Table 1:** Biometric and spirometric characteristics of the studied subjects.

	**Control** **(n = 28)**	**Smokers** **(n = 28)**	**p-value**
Male/Female	17/11	15/13	-
Age (years)	33.1 ± 8.2	35.1 ± 9.7	ns
Weight (kg)	66.1 ± 11.8	66.1 ± 10.7	ns
Height (cm)	167.4 ± 8.2	166.9 ± 7.9	ns
Pack-years	-	11.2 ± 7.3	-
FEV_1 _(L)	3.7 ± 0.7	3.5 ± 0.9	ns
FEV_1 _(%)	105.6 ± 11.8	105.1 ± 14.9	ns
FEF_25-75% _(L)	4.2 ± 1.3	3.8 ± 1.4	ns
FEF_25-75% _(%)	105.7 ± 27.2	100.9 ± 29.7	ns
FEV_1_/FVC (%)	85.0 ± 5.4	83.5 ± 6.4	ns
FEF/FVC (%)	95.7 ± 24.9	89.6 ± 29.6	ns

ns: non-significant; FEV_1_: forced expiratory volume in one second; FVC: forced vital capacity; FEF: forced expiratory flow; %: percentage of the predicted value. Pack-years was calculated multiplying the mean number of packs (one pack = 20 cigarettes) consumed daily by the number of years that the subject had their smoking habit.

**Figure 2:**
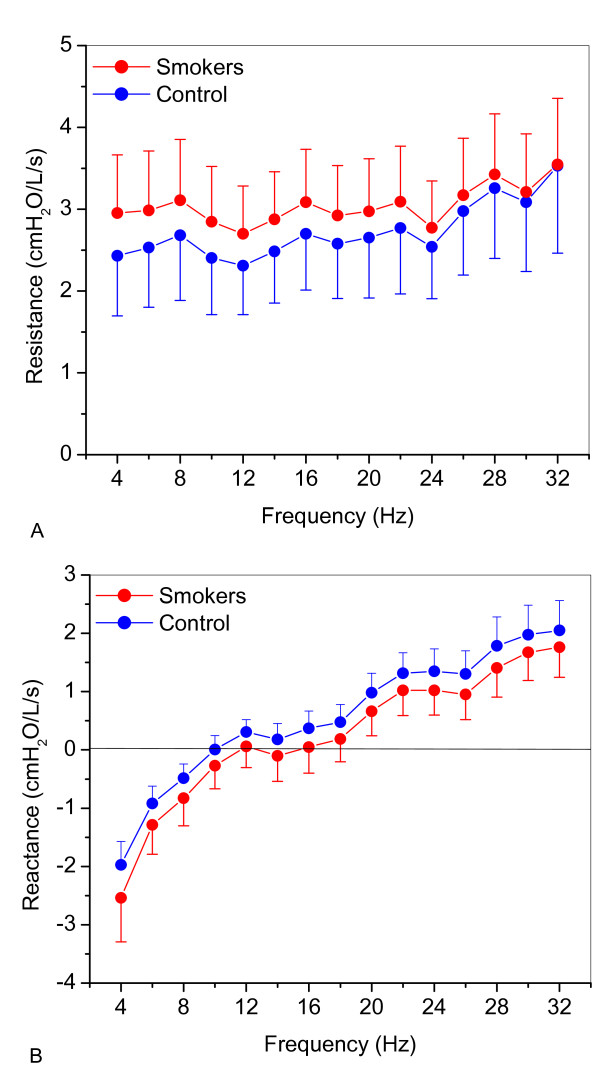
**Comparisons of the mean values of respiratory system resistance (A) and reactance (B) as a function of frequency in control and smoking subjects**.

The mean course and standard deviation of *Rrs *and *Xrs *as a function of frequency, in both control and smoking subjects, are presented in Figure 2. Smoking caused a uniform increase in the respiratory resistance curve, which was significantly different from the control curve (Figure 2A; p < 0.007). A non-significant decrease in *Xrs *(Figure 2B) was also observed, which resulted in a slight increase of the resonant frequency.

Table 2 describes the influence of smoking on FOT parameters. The mean values of *R0 *and *fr *presented highly significant increases in smoking patients (p < 0.001 and p < 0.002, respectively). The parameters *Rm *and *S *did not show significant changes in smokers. Similar comparisons revealed that significant decreases were observed comparing *Xm *(p < 0.0009) and *Crs, dyn *(p < 0.001). The absolute value of respiratory impedance was higher in smokers, and presented the most significant difference of the studied parameters (p < 0.0002).

**Table 2:** Forced oscillation parameters of the studied groups.

	**Control** **(n = 28)**	**Smokers** **(n = 28)**	**p-value**
R0 (cmH_2_O/L/s)	2.41 ± 0.80 2.10 - 2.72**	3.01 ± 0.75 2.72 - 3.30*	0.001
Rm (cmH_2_O/L/s)	2.47 ± 0.71 2.19 - 2.74**	2.96 ± 0.62 2.73 - 3.20*	ns
S (cmH2O/L/s^2^)	5.84 ± 14.44 0.24 - 11.44*	-4.67 ± 25.63 -14.61 - 5.27*	ns
fr (Hz)	10.88 ± 1.68 10.23 - 11.53**	14.18 ± 4.14 12.57 - 15.78**	0.002
Xm (cmH_2_O/L/s)	0.55 ± 0.27 0.44 - 0.65*	0.23 ± 0.40 0.07 - 0.38*	0.0009
Cdyn, rs (L/cmH_2_O)	0.021 ± 0.004 0.019 - 0.022*	0.017 ± 0.005 0.015 - 0.018*	0.001
Z4Hz (cmH_2_O/L/s)	3.14 ± 0.9 2.84 - 3.45**	3.96 ± 0.71 3.67 - 4.24*	0.0002

* Normal distribution; ** Non-normal distribution.

Values are presented as mean ± SD and confidence interval (95%).

**Figure 3:**
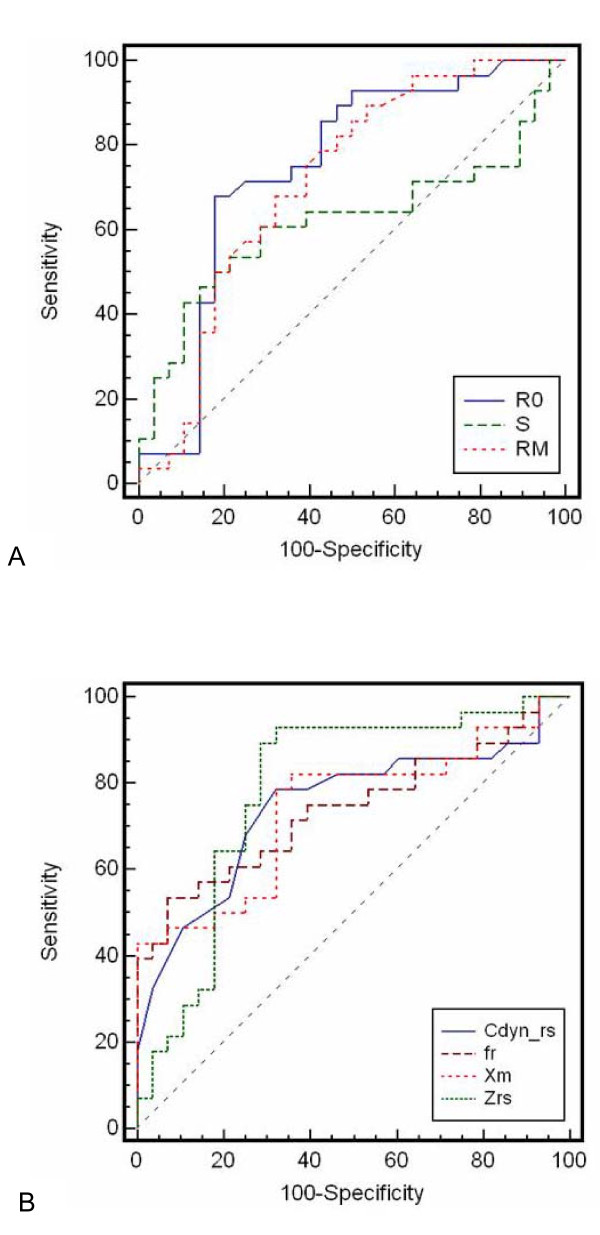
**Receiver operating characteristic (ROC) curves for resistive (A) and reactive (B) FOT indexes**. Derived parameters are described in Table 3.

Receiver operating characteristic curves for FOT and spirometric parameters are described in Figure 3. Derived parameters, sensitivity (Se), specificity (Sp), area under the curve (AUC) and the cut-off points are described in Table 3. The mean values of Se, Sp and AUC were significantly higher for the FOT parameters (p < 0.0001; p < 0.02, and p < 0.0001, respectively).

**Table 3:** Sensitivity (Se), specificity (Sp), area under the curve (AUC) and cut-off point of the Forced Oscillation and Spirometric parameters.

	**Se (%)**	**Sp (%)**	**AUC**	**Cut-off**
Forced Oscillation				
R0 (cmH_2_O/L/s)	71.4 (51.3-86.7)	75.0 (55.1-89.3)	0.75 (0.61-0.85)	2.70
S (cmH2O/L/s^2^)	64.3 (44.1-81.3)	60.7 (40.6-78.5)	0.62 (0.48-0.75)	2.66
Rm (cmH_2_O/L/s)	67.9 (47.7-84.1)	67.9 (47.7-84.1)	0.72 (0.58-0.83)	2.57
fr (Hz)	71.4 (51.3-86.7)	64.3 (44.1-81.3)	0.74 (0.61-0.85)	11.17
Xm (cmH_2_O/L/s)	78.6 (59.0-91.7)	67.9 (47.7-84.1)	0.74 (0.61-0.85)	0.42
Cdyn, rs (L/cmH_2_O)	78.6 (59.0-91.7)	67.9 (47.7-84.1)	0.75 (0.61-0.85)	0.018
Z4Hz (cmH_2_O/L/s)	75.0 (55.1-89.3)	75.0 (55.1-89.3)	0.79 (0.66-0.88)	3.58
Spirometry				
FEV_1_ (L)	57.1 (37.2-75.5)	60.7 (40.6-78.5)	0.58 (0.44-0.71)	3.60
FEV_1_ (%)	50.0 (30.7-69.3)	42.9 (24.5-62.8)	0.50 (0.36-0.64)	105.00
FEV_1_/FVC (%)	60.7 (40.6-78.5)	60.7 (40.6-78.5)	0.59 (0.45-0.72)	85.00
FEF_25-75%_ (L)	53.6 (33.9-72.5)	46.4 (27.5-66.1)	0.53 (0.40-0.67)	4.13
FEF_25-75%_ (%)	50.0 (30.7-69.3)	67.9 (47.7-84.1)	0.54 (0.40-0.68)	95.00
FEF/FVC (%)	60.7 (40.6-78.5)	60.7 (40.6-78.5)	0.55 (0.41-0.69)	94.50

Values are presented as mean and confidence interval (95%).

The differences in AUC of FOT and spirometric parameters are described in Table 4. In the studied sample, the FOT parameters always presented a higher AUC than the spirometric parameters (positive values in Table 4). This difference was significant in 26 of the 42 comparisons conducted (62%).

**Table 4:** Differences and statistical significance in the diagnostic performances of FOT and spirometric parameters, calculated by the difference between AUCs.

	**FEV_1_** **(L)**	**FEV_1_** **(%)**	**FEV_1_/FVC** **(%)**	**FEF_25-75%_** **(L)**	**FEF_25-75%_** **(%)**	**FEF/FVC** **(%)**
R0 (cmH_2_O/L/s)	0.17 ± 0.08*	0.25 ± 0.10*	0.16 ± 0.10	0.21 ± 0.09*	0.21 ± 0.10*	0.19 ± 0.10
S (cmH2O/L/s^2^)	0.04 ± 0.09	0.12 ± 0.10	0.03 ± 0.10	0.09 ± 0.10	0.08 ± 0.10	0.07 ± 0.10
Rm (cmH_2_O/L/s)	0.14 ± 0.08	0.22 ± 0.10*	0.13 ± 0.10	0.18 ± 0.09*	0.18 ± 0.10	0.16 ± 0.10
fr (Hz)	0.16 ± 0.08*	0.24 ± 0.09**	0.15 ± 0.09	0.21 ± 0.08*	0.20 ± 0.09*	0.19 ± 0.09*
Xm (cmH_2_O/L/s)	0.16 ± 0.09	0.24 ± 0.09**	0.15 ± 0.09	0.21 ± 0.09*	0.20 ± 0.09*	0.19 ± 0.09*
Crs, dyn (L/cmH_2_O)	0.17 ± 0.07*	0.25 ± 0.09**	0.16 ± 0.09	0.21 ± 0.08**	0.21 ± 0.09*	0.19 ± 0.10*
Z4Hz (cmH_2_O/L/s)	0.21 ± 0.08*	0.29 ± 0.10**	0.20 ± 0.10*	0.25 ± 0.09**	0.25 ± 0.10*	0.23 ± 0.10*

* p < 0.05; ** p < 0.01.

Positive values denotes higher values of AUC in the FOT parameters.

## Discussion

There is a general agreement in the literature that it is necessary to develop new accurate and noninvasive tests of lung function [2,40,41]. Recently, the National Heart Lung and Blood Institute recommended that research on new technologies to improve non-invasive testing of lung function in COPD should be a priority [42]. The FOT was suggested by Crapo et al. [6] as an attractive alternative for diagnosing obstruction in COPD, since it requires little patient effort and cooperation.

While many other published reports have used FOT to compare controls with ex-smokers and/or smoking subjects [5,15-17], to the best of our knowledge, this study is the first to investigate the performance of FOT in the detection of the effects of low levels of tobacco consumption. This study showed that FOT parameters detected important modifications after low tobacco consumption, which may be helpful in identifying patients at risk for COPD as early in the course of the disease as possible. This can contribute to prevent smoking uptake and maximize cessation, and thus, to prevent progress to clinically significant COPD.

### Biometric and spirometric characteristics of the studied groups

The two studied groups were of comparable age, weight and height, showing only non-significant statistical differences (Table 1). The study conducted by Verbank et al. [21] showed early changes in small airways in smokers with normal spirometry and as little as 10 pack-years of smoking history. The mean tobacco consumption of the studied smokers here was 11.2 ± 7.3 pack-years, characterizing a sample of smokers with early respiratory changes. In line with this hypothesis, we observed only small and non-significant reductions in the spirometric parameters of the smoking group (Table 1).

### Resistance and reactance *vs*. frequency curves

In line with previous results [5,15-17], we found that higher resistances are observed among smokers (Figure 2). A uniform increase was observed when comparing *Rrs *among the control group and smokers. This increase is consistent with that recently reported by Ionescu et al. [43] in a model study describing the effects of COPD on respiratory resistance. In close agreement with previously published results [15,16], we found that smokers displayed more negative *Xrs *values than healthy subjects (Figure 2B). Smoking provokes in its initial stages adverse reaction from the airway cells to the noxious components, which results in increases of small airways resistance. This is associated with a decrease in the dynamic compliance [44], which, in its turn, results in more negative values of Xrs. These results are associated with an increase in respiratory system non-homogeneity and in close agreement with the model study conducted by Ionescu et al. [43].

### Forced oscillation parameters

The significant increase in *R0 *(Table 2, p < 0.001) may be associated with airway obstruction [26]. Since it is known that the effects of smoking begin in the peripheral airways, we can speculate that this increase is associated with this section of the bronchial tree. The increase in *R0 *values for smoking subjects was consistent with previously published results [5,15-17,43] and suggests that *R0 *values could be useful to detect initial airway obstructions associated with smoking. In order to investigate this possibility, ROC curves were made (Figure 3). Although a variety of summary indices have been used to measure the performance of diagnostic systems [45,46], the area under the ROC curve (AUC) has the clinically useful interpretation of representing the probability of correctly discriminating between two subjects in a randomly selected pair of abnormal and normal subjects [39,47]. In the present study, 0.75 is considered the cut-off value for a useful discriminator. Thus, *R0 *reached acceptable values, identifying the respiratory changes due to a tobacco consumption near 10 pack-years with a sensitivity of 71.4% and a specificity of 75% (Table 3).

It is important to point out that it is possible that some people smoke for 10 years and do not actually have any structural change going on. Therefore, the results described in Figure 3 are limited to the studied sample. In spite of this limitation, these results confirmed that FOT can be used to detect the early alterations in the respiratory function presented in this sample due to the smoking process. This suggests that this technique could easily and non-invasively detect early airway obstruction in these patients.

In agreement with the present work, Hayes and colleagues [15] found no significant difference in *Rm *comparing non-smokers and smokers. This suggests that *Rm *would not be useful in detecting early changes due to smoking. Confirming this hypothesis, this parameter did not reach an AUC value that was considered useful for clinical use by the criteria used in this study (Figure 3, Table 3).

Although we can observe a clear difference in the *S *parameter between the controls and the smokers, this difference was not statistically significant (Table 2). It probably happens due to the high SD presented by this parameter. In agreement with these results, we observed a small AUC (Table 3), suggesting that this parameter may not be sufficiently sensitive to detect the early changes in the respiratory system of smoking patients.

Tobacco use was associated with a significant increase in *fr *(Table 2; p < 0.002). This links tobacco consumption with decreased respiratory system homogeneity and suggests that *fr *is useful as an index for the initial effects of smoking in the studied sample. The results described in Figure 3 and Table 3 shows that, although this parameter reached a borderline value of AUC, it may not be considered useful for the identification of the initial respiratory effects of smoking.

The low pack-year value of the smoking group resulted in decreased *Xm *mean values (Table 2, p < 0.0009). This reflects the impact of smoking on the reduction of respiratory system homogeneity and dynamic compliance of the studied subjects. The results described in Figure 3 and Table 3 show that, similar to *fr*, this parameter reached a borderline value of AUC, and may not be considered adequate.

Tobacco smoking resulted in a decline in *Crs, dyn *(Table 2, p < 0.001), which could be associated with an increase in peripheral airway resistance or with a reduction in the respiratory system compliance. Our results are in agreement with the findings of Verbank et al. [21] obtained in smokers with normal spirometry and as little as 10 pack-years of smoking history that indicated early changes of small airways in both the conductive and acinar lung zone compartments. In the studied sample, this parameter reached acceptable values for clinical use, identifying respiratory modifications with a sensitivity of 78.6% and a specificity of 67.9% (Figure 3 and Table 3).

As can be seen in Table 2, the modifications in resistive and elastic properties of smoking patients resulted in significant increases in *Z4Hz*. This parameter is related to the total mechanical load of the respiratory system. Therefore, it may be associated with fatigue and breathlessness in smoking patients. In agreement with the promising results of the initial analysis described in Table 2, we observed that this parameter achieved an adequate AUC to detect early respiratory alterations in the studied sample (0.79). In fact, *Z4Hz *was the most adequate measure to use to correctly identify the initial respiratory modifications in the studied smoking patients, with a sensitivity of 75% and a specificity of 75% (Table 3).

Recent recommendations for research in COPD [42] include the need for improved noninvasive mechanical tests of lung function. The present study was conducted as an effort to contribute in this direction, and showed that *R0*, *Crs, dyn *and *Z4Hz *are very promising parameters to non-invasively evaluate early modifications due to smoking. In the studied sample, these parameters were adequate to detect respiratory alterations even in conditions of small changes in spirometry. This suggests that the FOT test may be an useful screening tool in the management of smoking-induced lung disease, offering the possibility of showing abnormalities at a time when pathologic changes are still potentially reversible [21].

### Comparison between FOT and spirometry

According to Metz [38], the AUC is usually the best discriminator when we have a number of ROC curves to compare. In the studied groups, these analyses were clearly in favor of FOT parameters (Table 4). The results show that the AUC of *R0 *was significantly larger than that obtained for FEV_1_(L) and FEV_1_(%). These parameters are standard measures of lung function commonly used in the evaluation of patients with COPD [19]. The performance of *R0 *was also significantly higher than that of the FEF_25-75_(L) and FEF_25-75_(%), and borderline differences were achieved considering FEV_1_/FVC (%) and FEF/FVC (%).

There were no statistically significant differences in the AUC for the *S *parameter and the spirometric parameters, probably due to the high SD associated with these measurements. The diagnostic accuracy of *fr *and *Crs, dyn *was higher than all of the spirometric parameters except FEV_1_/FVC. Note that borderline differences were also achieved in these comparisons. On the other hand, the diagnostic performance of *Z4Hz *was higher than all of the spirometric parameters. These promising results suggest that *R0*, *fr*, *Crs, dyn *and *Z4Hz *values could be useful parameters in detecting early changes associated with smoking.

These results are in close agreement with previous studies that included asthmatic patients with normal spirometry, in which FOT parameters were useful to identify the initial respiratory modifications in these patients [25]. In more recent work, conducted in patients with sarcoidosis, it was observed that FOT parameters were adequate to detect respiratory alterations, even in conditions of normal spirometry [48].

### Limitations of the study

There are two potential limitations in the present study. First, we tested the hypothesis that, in a group of otherwise healthy current smokers, the forced oscillation technique was useful to detect the early effects of smoking on respiratory mechanics. The study was conducted comparing non-smoking and smoking subjects with low pack-years. A questionnaire was used as a reference in order to separate smokers and non-smokers. One could argue that a gold-standard technique was not used in this work to define the early changes in respiratory mechanics. However, as pointed out by Verbank et al. [21], in the early stages of smoking-induced lung disease, a gold-standard of peripheral lung damage is as yet impossible to obtain in human subjects. As it is widely known that small respiratory abnormalities are typical in subjects with low exposure to cigarette smoke [19], we compared the performance of FOT and spirometry in detecting these abnormalities in the same group of patients. It is important to point out that the respiratory changes observed in the present work are coherent with that obtained in a morphological model [43], confirming the consistency of these results.

The second limitation is that smoking history is difficult information to collect due to recall bias and changes in the amount of smoking throughout a lifetime. Thus it is possible that the value of pack-years in the smoking group is not very accurate. Since caution was taken to make FOT and spirometric measurements in the same subjects, this uncertainty equally affects the two measures. Therefore, this uncertainty is not a problem in the present study.

## Conclusion

In summary, this study has shown that the FOT provided parameters that can be used to detect early alterations in respiratory mechanics related to smoking. *R0*, *Crs, dyn *and *Z4Hz *were the most adequate parameters for the detection of these respiratory changes.

The comparison of the accuracy of FOT and spirometric parameters indicated that, in general, forced oscillation parameters were more accurate than spirometric indices to identify small alterations due to smoking.

These results suggest that the FOT can be proposed as a complementary method to detect the harmful effects of smoking while they are still potentially reversible, contributing to the prevention of COPD development. We hope that this study can help the legion of smoking patients that seeks pulmonary laboratories for the solution of breathing disorders.

## Competing interests

The authors declare that they have no competing interests.

## Authors' contributions

ACDF conducted the measurements for this study, analyzed the data, and drafted the manuscript. AJL provided data and subject identification. JMJ mentored ACDF and participated in data analysis process. PLM mentored ACDF, organized the study and helped to draft the manuscript. All authors have read and approved this manuscript.
